# Prevalence of Thyroid Dysfunction Among Greek Type 2 Diabetic Patients Attending an Outpatient Clinic

**DOI:** 10.4021/jocmr2010.03.281w

**Published:** 2010-03-25

**Authors:** Athanasia Papazafiropoulou, Alexios Sotiropoulos, Anthi Kokolaki, Marina Kardara, Petroula Stamataki, Stavros Pappas

**Affiliations:** a The Third Department of Internal Medicine and Center of Diabetes, General Hospital of Nikaia Ag. Panteleimon, Piraeus, Greece

## Abstract

**Background:**

The aim of the present study was to determine the prevalence of thyroid dysfunction in patients with type 2 diabetes (T2D) attending an outpatient clinic.

**Methods:**

We examined thyroid dysfunction in a total of 1,092 patients with T2D.

**Results:**

Prevalence rate of thyroid dysfunction was 12.3%. In the group with thyroid dysfunction there was an excess of females in comparison with the group without thyroid dysfunction (P < 0.001). In addition, patients with thyroid dysfunction had higher values of body mass index (P = 0.03) and HDL-cholesterol levels (P = 0.01), and lower values of LDL-cholesterol levels (P = 0.001) in comparison with patients without thyroid dysfunction. Multivariate analysis demonstrated that presence of thyroid dysfunction was related with gender (OR: 0.220, 95% CI: 0.141 - 0.352) and LDL-cholesterol levels (OR: 0.990, 95% CI: 0.985 - 0.995).

**Conclusions:**

The prevalence of thyroid dysfunction among Greek diabetic patients is 12.3%. Diabetic women were more frequently affected than men. Presence of thyroid dysfunction was associated with lower levels of LDL-cholesterol concentrations.

**Keywords:**

Type 2 diabetes mellitus; Thyroid dysfunction; Hypothyroidism; Gender; LDL-cholesterol; Greece

## Introduction

The first reports showing the association between diabetes and thyroid dysfunction were published in 1979 [1, 2]. Since then a lot of studies in different countries have tried to estimate the prevalence of thyroid dysfunction among diabetic patients [1-5]. The reported prevalence of thyroid dysfunction in diabetes varying from 2.2 to 17% [1-4]. Another study reported high prevalence of abnormal TSH concentration in patients with type 2 diabetes (T2D) (31%) [5]. In addition, diabetic women are more frequently affected than men and hypothyroidism is more common than thyrotoxicosis [5]. It has been showed that subclinical hypothyroidism affects almost one in 20 women with T2D [5].

To the best of our knowledge no studies have been done to estimate the prevalence of thyroid dysfunction in type 2 diabetic patients in our country. Therefore, the aim of the present study was to determine the prevalence of thyroid dysfunction in patients with T2D attending an outpatient clinic.

## Patients and Methods

### Subjects

We examined a total of 1,092 patients with T2D attending the diabetes outpatient clinic of our hospital from January 2008 to June 2009. Diagnosis of diabetes was based on the American Diabetes Association criteria [6]. Patients with at least three visits the last year were enrolled in the study. A medical history, regarding the age at diagnosis of diabetes, the presence of cardiovascular disease, the presence of diabetic complications, and current medication, was obtained. Patients who reported taking T4, T3, carbimazole, methimazole or propylthiouracil, and those with a history of thyroidectomy, radioactive iodine treatment, were identified as having thyroid dysfunction.

The study protocol was approved by the Scientific and Ethical Committee of the General Hospital of Nikaia. Full informed written consent was obtained from all patients.

### Methods

Blood samples were drawn after 10 - 12 hours fast, for measurement of plasma HbA_1c_ and lipid profile. Body weight with subjects in light clothing without shoes and height was measured and body mass index (BMI) was calculated. Blood pressure was recorded as the mean of three consecutive measurements in the sitting position taken 5 min apart. Hypertension was defined according to the current guidelines [7] as BP levels ≥ 140/90 mm Hg or the use of anti-hypertensive drugs.

Coronary artery disease (CAD) was defined as presence of angina, history of previous myocardial infarction, positive stress testing, revascularization procedures or stenosis > 50% at the coronary arteries. The renal status was based on the albumin excretion rate (AER) measured in at least two out of three consecutive 24-h timed urine collections. Patients were classified as normo- (AER < 20 mg/min), micro- (AER 20 - 199 mg/min), or macroalbuminurics (AER > 200 mg/min). Direct fundoscopy was performed in all patients through dilated pupils.

### Statistical analysis

Statistical analysis was preformed using programs available in the SPSS statistical package (SPSS 15.0, Chicago, USA). All variables were tested for normal distribution of the data. Data are shown as mean ± SD, unless it is stated otherwise. A two sample t-test was used to assess differences in continuous variables, while a chi-square test was used for categorical variables. Univariate binary logistic analysis was performed to look for the relationship between thyroid dysfunction and the variables of interest in the sample population. Then, multivariate analysis was performed (backward stepwise method) to look for independent associations between thyroid dysfunction and the variables of interest. All independent variables in the multivariate analysis were tested for multicolinearity. P < 0.05 was considered statistically significant.

## Results

Prevalence rate of thyroid dysfunction was 12.3% in the study group. The clinical and laboratory characteristics of the study patients according to the presence or absence of thyroid dysfunction are showed in Table 1.

Diabetic patients with and without thyroid dysfunction did not differ in terms of age and duration of diabetes. However, in the group of diabetic patients with thyroid dysfunction there was an excess of females in comparison with the group without thyroid dysfunction (78.4 vs. 48.3 %, P < 0.001, respectively). In addition, diabetic patients with thyroid dysfunction had higher values of BMI (31.76 ± 5.67 vs. 30.65 ± 5.47 Kg/m^2^, P=0.03, respectively) and plasma HDL-cholesterol levels (51.70 ± 20.45 vs. 47.35 ± 18.91 mg/dl, P = 0.01, respectively), and lower values of plasma LDL-cholesterol levels (114.94 ± 41.48 vs. 128.05 ± 42.87 mg/dl, P = 0.001, respectively) in comparison with the diabetic patients without thyroid dysfunction (Table 1).

The univariate logistic analysis showed significant associations between the presence of thyroid dysfunction and gender [male vs. female, odds ratio (OR): 0.250, 95% Confidence Intervals (95% CI): 0.168-0.390], BMI (OR: 1.036, 95% CI: 1.004 -1.069), LDL-cholesterol levels (OR: 0.992, 95% CI: 0.987 - 0.997) and HDL-cholesterol levels (OR: 1.008, 95% CI: 1.000 - 1.017). No any significant relationships were found between presence of thyroid dysfunction and age, duration of diabetes, blood pressure, microvascular and macrovascular complications (Table 2).

Multivariate analysis demonstrated, after controlling for BMI, that presence of thyroid dysfunction was related only with gender (OR: 0.220, 95% CI: 0.141 - 0.352) and LDL-cholesterol levels (OR: 0.990, 95% CI: 0.985 - 0.995) (Table 2).

**Table 1:** Clinical and Laboratory Characteristics of Diabetic Patients According to the Presence of Thyroid Dysfunction

	Diabetic subjects with thyroid dysfunction	Diabetic subjects without thyroid dysfunction	P
N	134	958	
Age (yrs)	65.53 ± 11.77	67.07 ± 12.11	0.17
Males/females n (%)	29 (21.6)/105 (78.4)	495 (51.7)/463 (48.3)	< 0.001
Body mass index (Kg/m^2^)	31.76 ± 5.67	30.65 ± 5.47	0.03
Duration of diabetes (yrs)	14.28 ± 9.55	14.64 ± 9.66	0.68
Systolic blood pressure (mm Hg)	137.46 ± 18.42	134.73 ± 18.20	0.11
Diastolic blood pressure (mmHg)	77.87 ± 10.41	78.63 ± 10.05	0.41
HbA_1c_ (%)	7.38 ± 1.57	7.81 ± 1.69	0.18
Total cholesterol (mg/dl)	199.80 ± 98.30	207.24 ± 57.31	0.15
HDL cholesterol (mg/dl)	51.70 ± 20.45	47.35 ± 18.91	0.01
LDL cholesterol (mg/dl)	114.94 ± 41.48	128.05 ± 42.87	0.001
Triglycerides (mg/dl)	161.41 ± 119.72	164.39 ± 103.54	0.76
Hypertension (yes) n (%)	85 (63.4)	553 (57.7)	0.21
Dyslipidemia (yes) n (%)	70 (52.2)	439 (45.8)	0.16
Retinopathy (yes) n (%)	27 (20.1)	171 (17.9)	0.52
Nephropathy (yes) n (%)	-	39 (4.1)	-
Coronary artery disease (yes) n (%)	23 (17.2)	165 (17.3)	0.98
Treatment for diabetes n (%)			
Antidiabetic tablets	85 (63.4)	628 (65.6)	0.63
Insulin	47 (35.1)	323 (33.7)	0.75

**Table 2:** Univariate and Multivariate Logistic Analysis: The Association Between Various Parameters With Thyroid Dysfunction in T2D Patients

	Univariate analysis			Multivariate analysis		
	Odds ratio	95% Confidence Intervals	P-value	Odds ratio	95% Confidence Intervals	P-value
Gender	0.250	0.168 - 0.390	< 0.001	0.222	0.141 - 0.352	< 0.001
BMI	1.036	1.004 - 1.069	0.03	-	-	-
LDL- cholesterol	0.992	0.987 - 0.997	0.001	0.990	0.985 - 0.995	< 0.001

## Discussion

The present study showed that in a sample of Greek diabetic patients the prevalence of thyroid dysfunction was 12.3%. The above results are in agreement with previous studies showing an association between T2D and thyroid dysfunction [1-5]. A study by Smithson et al. showed a prevalence of 10.8% of thyroid dysfunction in diabetic patients registered in general practice [4]. Another study by Perros et al. in a randomly selected group of 1,310 diabetic adults estimated that the prevalence of thyroid dysfunction was found 13.4% [3]. A recent study reported that thyroid dysfunction was present in 16% of Saudi T2D patients [8]. Also, a study in Jordan showed that the overall prevalence of thyroid dysfunction was 12.5% in T2D patients [9].

In our study, we reported a higher prevalence of thyroid dysfunction among diabetic females. It is well established that hypothyroidism is more common in diabetic females. In a study by Perros et al. the prevalence of thyroid dysfunction was 10.9% in females and 6.9% in males [3]. The NHANES III study reported that the prevalence of subclinical hypothyroidism was 3.4% in males and 5.8% in females [10]. In addition, a study in 420 adult females with T2D randomly selected from participants in the community-based Fremantle Diabetes Study showed that the prevalence of subclinical hypothyroidism was 8.6% [11]. Finally, a recent study revealed that the prevalence of subclinical hypothyroidism was 5.2% in males and 8.4% in females with T2D [12].

In the present study we found that diabetic patients with thyroid disorders had better lipid profile, regarding LDL- and HDL-cholesterol levels, compared with diabetic subjects without thyroid dysfunction. However, the existing literature data regarding the association between lipids and thyroid function are controversial. While dyslipidemia is a reported complication of overt hypothyroidism in nondiabetic [13-15] and diabetic [16] subjects, a recent meta-analysis [17] has showed that subclinical hypothyroidism does not seem to be associated with dyslipidemia. A study by Chubb et al. [11] did not find any significant relationship between subclinical hypothyroidism and the presence of dyslipidemia. Also, in large studies of subclinical hypothyroidism and coronary heart disease [18-20], there was no association with raised serum cholesterol. An explanation of our findings was that all the participants were under thyroid hormone replacement therapy, which has been showed to improve serum lipids, in particular LDL-cholesterol levels [21, 22].

In conclusion, the present study showed that the prevalence of thyroid dysfunction among Greek diabetic patients attending an outpatient clinic was 12.3%. Diabetic women were more frequently affected than men. In addition, presence of thyroid dysfunction was associated with lower levels of LDL-cholesterol. However, as data were collected from a referral tertiary center of diabetes, they can not be extrapolated to the total population. Therefore, larger epidemiological studies are needed to estimate the prevalence of thyroid dysfunction in Greek T2D patients.

## References

[1] Feely J, Isles TE (1979). Screening for thyroid dysfunction in diabetics. Br Med J.

[2] Gray RS, Irvine WJ, Clarke BF (1979). Screening for thyroid dysfunction in diabetics. Br Med J.

[3] Perros P, McCrimmon RJ, Shaw G, Frier BM (1995). Frequency of thyroid dysfunction in diabetic patients: value of annual screening. Diabet Med.

[4] Smithson MJ (1998). Screening for thyroid dysfunction in a community population of diabetic patients. Diabet Med.

[5] Celani MF, Bonati ME, Stucci N (1994). Prevalence of abnormal thyrotropin concentrations measured by a sensitive assay in patients with type 2 diabetes mellitus. Diabetes Res.

[6] (1997). Report of the Expert Committee on the Diagnosis and Classification of Diabetes Mellitus. Diabetes Care.

[7] Chobanian AV, Bakris GL, Black HR, Cushman WC, Green LA, Izzo JL, Jones DW (2003). Seventh report of the Joint National Committee on Prevention, Detection, Evaluation, and Treatment of High Blood Pressure. Hypertension.

[8] Akbar DH, Ahmed MM, Al-Mughales J (2006). Thyroid dysfunction and thyroid autoimmunity in Saudi type 2 diabetics. Acta Diabetol.

[9] Radaideh AR, Nusier MK, Amari FL, Bateiha AE, El-Khateeb MS, Naser AS, Ajlouni KM (2004). Thyroid dysfunction in patients with type 2 diabetes mellitus in Jordan. Saudi Med J.

[10] Hollowell JG, Staehling NW, Flanders WD, Hannon WH, Gunter EW, Spencer CA, Braverman LE (2002). Serum TSH, T(4), and thyroid antibodies in the United States population (1988 to 1994): National Health and Nutrition Examination Survey (NHANES III). J Clin Endocrinol Metab.

[11] Chubb SA, Davis WA, Inman Z, Davis TM (2005). Prevalence and progression of subclinical hypothyroidism in women with type 2 diabetes: the Fremantle Diabetes Study. Clin Endocrinol (Oxf).

[12] Chen HS, Wu TE, Jap TS, Lu RA, Wang ML, Chen RL, Lin HD (2007). Subclinical hypothyroidism is a risk factor for nephropathy and cardiovascular diseases in Type 2 diabetic patients. Diabet Med.

[13] Staub JJ, Althaus BU, Engler H, Ryff AS, Trabucco P, Marquardt K, Burckhardt D (1992). Spectrum of subclinical and overt hypothyroidism: effect on thyrotropin, prolactin, and thyroid reserve, and metabolic impact on peripheral target tissues. Am J Med.

[14] Elder J, McLelland A, O'Reilly DS, Packard CJ, Series JJ, Shepherd J (1990). The relationship between serum cholesterol and serum thyrotropin, thyroxine and tri-iodothyronine concentrations in suspected hypothyroidism. Ann Clin Biochem.

[15] Johnston J, McLelland A, O'Reilly DS (1993). The relationship between serum cholesterol and serum thyroid hormones in male patients with suspected hypothyroidism. Ann Clin Biochem.

[16] Gray RS, Smith AF, Clarke BF (1981). Hypercholesterolemia in diabetics with clinically unrecognised primary thyroid failure. Horm Metab Res.

[17] Hueston WJ, Pearson WS (2004). Subclinical hypothyroidism and the risk of hypercholesterolemia. Ann Fam Med.

[18] Tunbridge WM, Evered DC, Hall R, Appleton D, Brewis M, Clark F, Evans JG (1977). Lipid profiles and cardiovascular disease in the Whickham area with particular reference to thyroid failure. Clin Endocrinol (Oxf).

[19] Hak AE, Pols HA, Visser TJ, Drexhage HA, Hofman A, Witteman JC (2000). Subclinical hypothyroidism is an independent risk factor for atherosclerosis and myocardial infarction in elderly women: the Rotterdam Study. Ann Intern Med.

[20] Imaizumi M, Akahoshi M, Ichimaru S, Nakashima E, Hida A, Soda M, Usa T (2004). Risk for ischemic heart disease and all-cause mortality in subclinical hypothyroidism. J Clin Endocrinol Metab.

[21] Ganotakis ES, Mandalaki K, Tampakaki M, Malliaraki N, Mandalakis E, Vrentzos G, Melissas J (2003). Subclinical hypothyroidism and lipid abnormalities in older women attending a vascular disease prevention clinic: effect of thyroid replacement therapy. Angiology.

[22] Becerra A, Bellido D, Luengo A, Piedrola G, De Luis DA (1999). Lipoprotein(a) and other lipoproteins in hypothyroid patients before and after thyroid replacement therapy. Clin Nutr.

